# fNIRS Evaluation of Frontal and Temporal Cortex Activation by Verbal Fluency Task and High-Level Cognition Task for Detecting Anxiety and Depression

**DOI:** 10.3389/fpsyt.2021.690121

**Published:** 2021-06-29

**Authors:** Xuenan Lang, Dan Wen, Qiqi Li, Qin Yin, Mingyu Wang, Yong Xu

**Affiliations:** ^1^First Hospital of Shanxi Medical University, Taiyuan, China; ^2^Department of Psychiatry, First Hospital/First Clinical Medical College of Shanxi Medical University, Taiyuan, China; ^3^Department of Mental Health, Shanxi Medical University, Taiyuan, China

**Keywords:** functional near-infrared spectroscopy, high-level cognition task, verbal fluency task, anxiety, depression

## Abstract

Anxiety and depression are widespread psychosis which are believed to affect cerebral metabolism, especially in frontal and temporal cortex. The comorbidity patients of anxiety and depression (A&D) have more serious clinical symptoms. Functional near-infrared spectroscopy (fNIRS) is a noninvasive modality used to monitor human brain oxygenation, and it could be considered as a potential tool to detect psychosis which may lead to abnormal cerebral oxygen status when the brain is activated. However, how sensitive the cerebral oxygenation response to the cortex activation and whether these responses are consistent at different stages of A&D or different regions still remains unclear. In this study, a conventional physiological paradigm for cortex activation, i.e., verbal fluency task (VFT), and a relatively new paradigm, i.e., high-level cognition task (HCT), were compared to detect A&D through a longitudinal measurement of cerebral oxygen status by fNIRS. The A&D patients at the acute, consolidation and maintenance stages as well as the healthy subjects participated in the VFT and HCT paradigms, respectively. For the VTF paradigm, the subject was instructed to answer questions of phrase constructions within 60 s. For the HCT paradigm, the subject was instructed to categorize items, logical reasoning, and comprehensive judgment and write down the answers within 60 s. For most of the subjects, the oxy-Hb is found to increase remarkably, accompanied with a relatively small reduction in deoxy-Hb when subject to both paradigms. The statistical analyses show a relatively large variability within any group, leading to the significant difference that was only found between A&D at the acute stage and healthy subjects in the temporal lobe region (*p* < 0.001). Nevertheless, HCT would activate more oxygen increment when compared with the VFT, with a large integral value in oxy-Hb. On average, the oxy-Hb integral value of the A&D patients differs substantially at different stages when subject to HCT paradigm. Moreover, the prefrontal lobe and temporal lobe responses were more consistent to the HCT paradigm rather than the VFT paradigm. Under the VFT paradigm, however, no remarkable difference in integral value was found among the three stages, either at the prefrontal lobe or at the temporal lobe. This study indicated that HCT, which is intensively involved in brain function, would activate more oxygenation changes in the cerebral cortex. Additionally, with good performance at distinguishing different stages according to the oxy-Hb criterion, the HCT has the potential to evaluate the therapeutic effects for A&D patients.

## Background

It is well-known that the human brain controls emotions and cognitive activities. The prefrontal lobe of the brain is associated with the formation and expression of language. If the prefrontal lobe is damaged, these abilities will be significantly impaired. The temporal lobe of the brain is associated with autonomous consciousness; therefore, it is critical in language comprehension, hearing, visual processing, and facial recognition. Cognition is a major determinant of social function in patients with psychosis (1, 2).

Anxiety disorders and depression disorders are widespread psychiatric diseases. According to the WHO World Mental Health Survey, the comorbidity rate between anxiety and depression was 45.7% (3). The comorbidity patients (anxiety and depression, A&D) have a worse overall prognosis and more severe functional impairment (4, 5). Follow-up studies have found that, compared with major depressive disorder (MDD), A&D has a unique symptomatology that differs in cognitive control and emotion responses. A&D has a declined cognitive control regulation but enhanced emotion responses; MDD has declined cognitive control regulation and delayed emotion responses (6). Related studies support the possibility that A&D could be an independent psychosis.

It is difficult to diagnose psychosis through anatomical abnormalities by using regular brain imaging techniques such as MRI or CT. The symptoms of psychosis are often associated with brain function rather than anatomy or morphology. The functional near-infrared spectroscopy (fNIRS) technique is a non-invasive technique developed in recent years to assess brain function. fNIRS is capable of real-time acquisition of cerebral oxygen signals as well as dynamic monitoring of physiological and pathological processes in the cerebral cortex. Therefore, fNIRS has been widely used to assess brain function in psychosis (7, 8). In addition, the cerebral oxygen metabolism would elevate when performing brain-loaded cognitive tasks. The fNIRS technique measures the characteristics of cortex oxyhemoglobin and deoxyhemoglobin changes, which indirectly reflect the function of the brain.

The fNIRS has been utilized by numerous laboratories to design task models, including oral fluency and working memory tasks (9–11), which have been found to be associated with prefrontal and temporal activation. Neural activities within the dorsolateral prefrontal cortex (DLPFC) reflect the emotion responses, especially to negative stimulation. Post-treatment activation of the DLPFC would also enhance emotion controls. The activity of DLPFC in patients with depression decreased not only in cognitive tasks, such as the Stroop task, the emotional oddity task, the go/no-go task, and the self-judgment task (12–15), but also in emotion recognition tasks (16–19). The DLPFC has executive functions which are parts of cognitive abilities and contribute to predictions of future behavior consequences.

The activation of the frontal and temporal lobes has been repeatedly proven through tasks (20). At present, the most widely used cognitive task is the verbal fluency task (VFT). With less often adoption, the high-level cognition task (HCT) reflects the advanced thinking activity of the brain. High-level cognition refers to the formation of thought, such as conception, abstraction, judgment, reasoning, and execution, also known as rational cognition (21). Conceptual classification means categorizing things according to their different properties, such as agricultural products, animals, fruits, and so on. Critical thinking belongs to judgment and reasoning, such as the evaluation of processes, results, and methods (22). Execution ability is to solve problems or plan for the future through cognitive resources (23). High-level cognition is the most complex (24). Both VFT and HCT are advanced cognitive tasks. The verbal fluency task reflects the ability to pick up words. The high-level cognition task reflects the abilities of judgment, conception, and reasoning. VFT is to extract related concepts, while HCT is not only to extract related concepts but also to integrate and process the concepts, which has a high degree of complexity. In this study, the subjects were asked to define a concept, such as tree, student, dolphin, apple, chef, eggplant, and dog, and then categorize the concept and classify it, such as plant, occupation, animal, and fruit. Next, the subjects were asked to rate the difficulty of the question. Studies have shown that cognitive impairment of psychosis persists from the onset to remission stage (25), including declines in attention, memory, and executive function (26, 27). The HCT might be more appropriate for differentiating psychiatric disorders when compared with the conventional VFT. Thus far, the comprehensive comparison between VFT and HCT has not been conducted, which is the main goal of this study (28, 29).

In this study, we conducted the first attempt to extensively evaluate the VFT and HCT in the A&D population by using fNIRS. Based on the subject's knowledge background, a comprehensive assessment of cognitive abilities, including attention, memory, and discrimination, calculation, categorization, logical thinking, and other rational thinking abilities, was conducted. The subjects were required to apply multiple types of abilities in the HCT. The fNIRS was used to assess the differences between VFT and HCT, and data from the prefrontal and temporal lobes of the brain were collected and analyzed for comparison. The derived outcomes were used to explore the task that can assess cerebral cortex function in A&D and provide aid in clinical diagnosis and intervention.

## Materials and Methods

### Participants

According to the Diagnostic and Statistical Manual of Mental Disorders-V (DSM-V) standard, the patients were diagnosed with depressive disorder and suffering from anxiety (30). The diagnosis was determined by three experienced physicians. From the outpatient or inpatient divisions of the Department of Psychiatric Health, First Hospital of Shanxi Medical University, we recruited 30 patients with A&D at the acute stage, consolidation stage, and maintenance stage, with 10 subjects in each group. In addition, 10 age-matched healthy controls (HCs), none of whom had a family history of psychosis, were also recruited. Those who have a history of neurological or other psychosis, substance abuse, severe medical illness, or cognitive dysfunction were excluded. All of the subjects were between 15 and 55 years of age. The four groups of subjects (i.e., HCs and the A&D at the acute, consolidation, and maintenance stages) participated in the VFT and HCT paradigm, respectively, resulting in a total of eight sets of hemodynamic data, with 10 data in each set. This study was approved by the Ethics Committee of the First Hospital of Shanxi Medical University, and informed consent was obtained from each participant. All the subjects were native Chinese speakers with speaking and reading ability, had an educational background of high school or above, and were able to cooperate effectively with this study.

### Clinical Assessments

For A&D, clinical assessment included age, duration of illness, intake of psychiatric medicine, and duration of medication. For A&D and HCs, the clinical symptoms were assessed using the Hamilton Depression Scale (HAMD-24) and the Hamilton Anxiety Scale (HAMA-14).

### VFT Task and HCT Task

The task instructors were psychiatric clinicians with well-training. Guided by the instructor, it took ~160 s for A&D and HC subjects to complete the VFT. During the 30-s baseline period, the subjects sat still in a chair and were required to repeat counting from one to five until the task began. During the 60-s task period, the subjects were asked to construct as many phrases as possible using simple words such as sky, earth, and big. At the end of the phrase constructions, the subjects were asked to repeat counting from one to five until the end of the task, which took 70 s. The subjects then rested for 5 min, and then the HCT was started. The detailed procedures for the HCT paradigm are depicted elsewhere (25–27). The subjects sat still in a chair and wrote down the answers on a paper for a few questions during the 60-s period. In this study, the subjects were asked to define a concept and classify it, such as tree, student, dolphin, apple, chef, eggplant, and dog, and then categorize the concept, such as plant, occupation, animal, and fruit. Next, the subjects were asked to rate the difficulty of the question. Prior to the tasks, the subjects were asked to repeat counting from one to five for a baseline period of 30 s until the task began. The task period took 60 s, and the subjects would repeat counting until the end of the task, which took 70 s. The subjects were asked to write down as many answers as possible. After the task was completed, the correct rate of answers was recorded.

### fNIRS Detection

The principle of fNIRS is based on the absorption and scattering of near-infrared light in cerebral tissue. Photons are absorbed or scattered by hemoglobin in the tissue, causing attenuation of the light; thus, the hemoglobin content is estimated according to the modified Beer–Lambert law. The 3 × 11 probe holder was placed on the scalp surface, and the lowest channel was positioned at Fp1–Fp2 according to the international 10–20 system. The detection sites mainly covered the prefrontal and bilateral temporal lobe regions (31, 32), and the distance between two adjacent source-detector fibers was set at 3.0 cm, with the covering region between the source-detectors defined as “channel.” The prefrontal lobe covered 18 channels, and the left and right temporal lobes covered 34 channels. The sampling rate of the fNIRS is 10 Hz.

A 52-channel fNIRS system (ETG-4100, Hitachi Medical Systems, Japan) was utilized to longitudinally monitor the oxygenation changes throughout the VFT or HCT paradigm. The fNIRS measurements include a 30-s baseline, a 60-s task (VFT or HCT), and a 70-s post-task recovery. The cerebral oxygenation variables measured from the fNIRS system, including the changes in oxy- and deoxy-hemoglobin (i.e., oxy-Hb and deoxy-Hb), were obtained. Moreover, a derived parameter, namely, integral value, was calculated from these oxygenation variables. Specifically, the integral value is defined as the sum of hemodynamic concentration (oxy-Hb or deoxy-Hb) from task beginning to task ending (i.e., 60-s task period).

### Data Analysis

One-way ANOVA was used to test the significant difference of hemodynamic data among the four groups (i.e., HCs and A&D patients at the acute, consolidation, and maintenance stages). Regression analysis was performed to investigate the influence of age on hemodynamic data. *p* < 0.05 was considered significant for all statistical results.

## Results

### Age-Related Changes in VFT and HCT

Table 1 shows that age has no influence on the oxy-Hb and deoxy-Hb data (*r* < 0.1, *p* > 0.05) for both VFT and HCT paradigms.

**Table 1:** Standard deviation of age.

**Stage**	**Mean**	**Standard deviation**	**Probability**
Acute	60.4	18.2	
Consolidation	57.5	10.3	
Maintenance	61.6	17.9	
Healthy controls	32.1	10.6	>0.05

### The Activation of HCT and VFT Tasks in the Prefrontal Lobe

**Figure 1:**
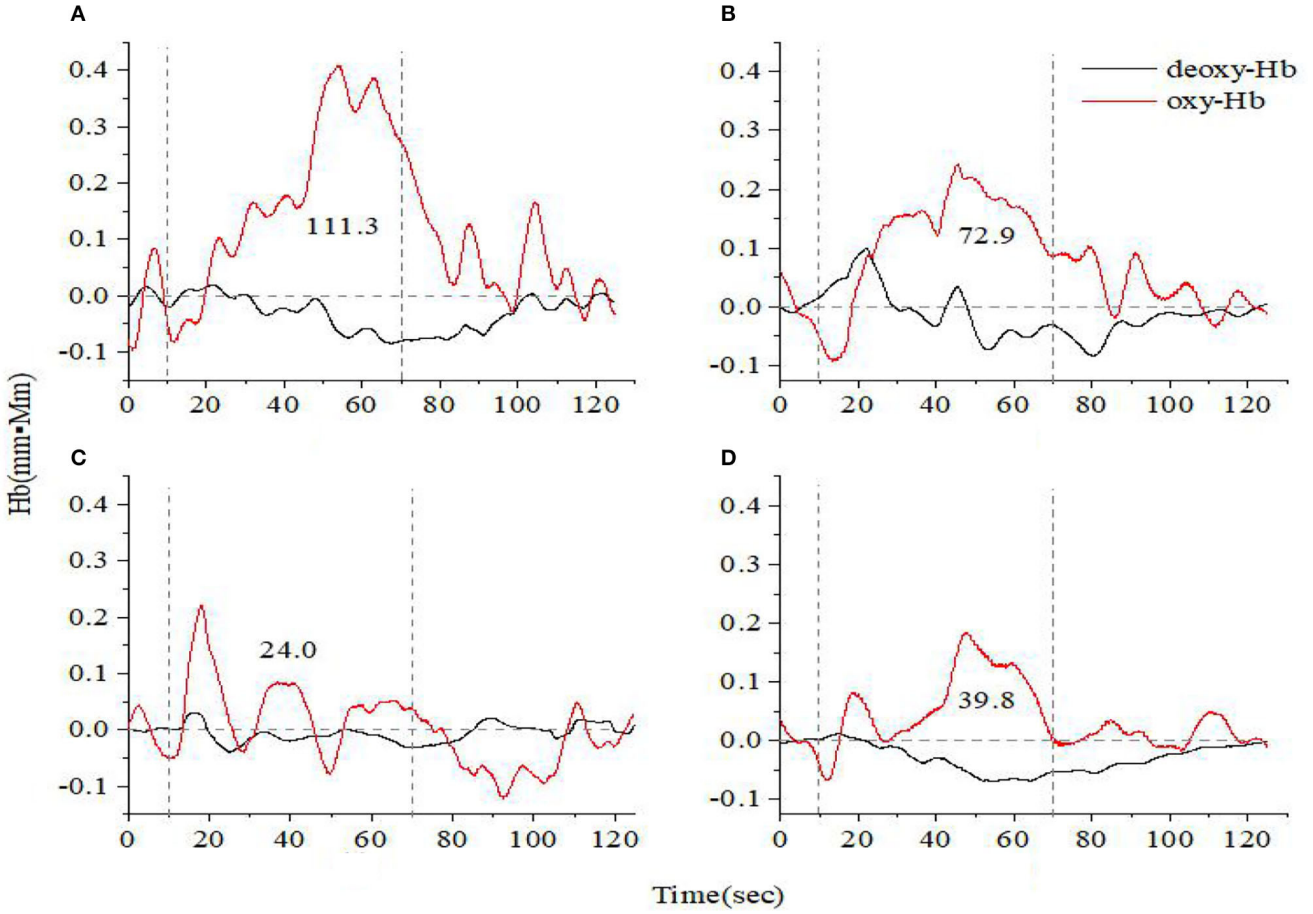
Time course of hemoglobin concentration changes in a representative healthy control **(A)** and a representative anxiety and depression patient at the acute **(B)**, consolidation **(C)**, and maintenance stages **(D)**. All of them completed the verbal fluency task. The red line indicates the amount of change in oxyhemoglobin concentration (oxy-Hb), and the black line indicates the amount of change in deoxyhemoglobin concentration (deoxy-Hb). The numbers indicate integral values.

**Figure 2:**
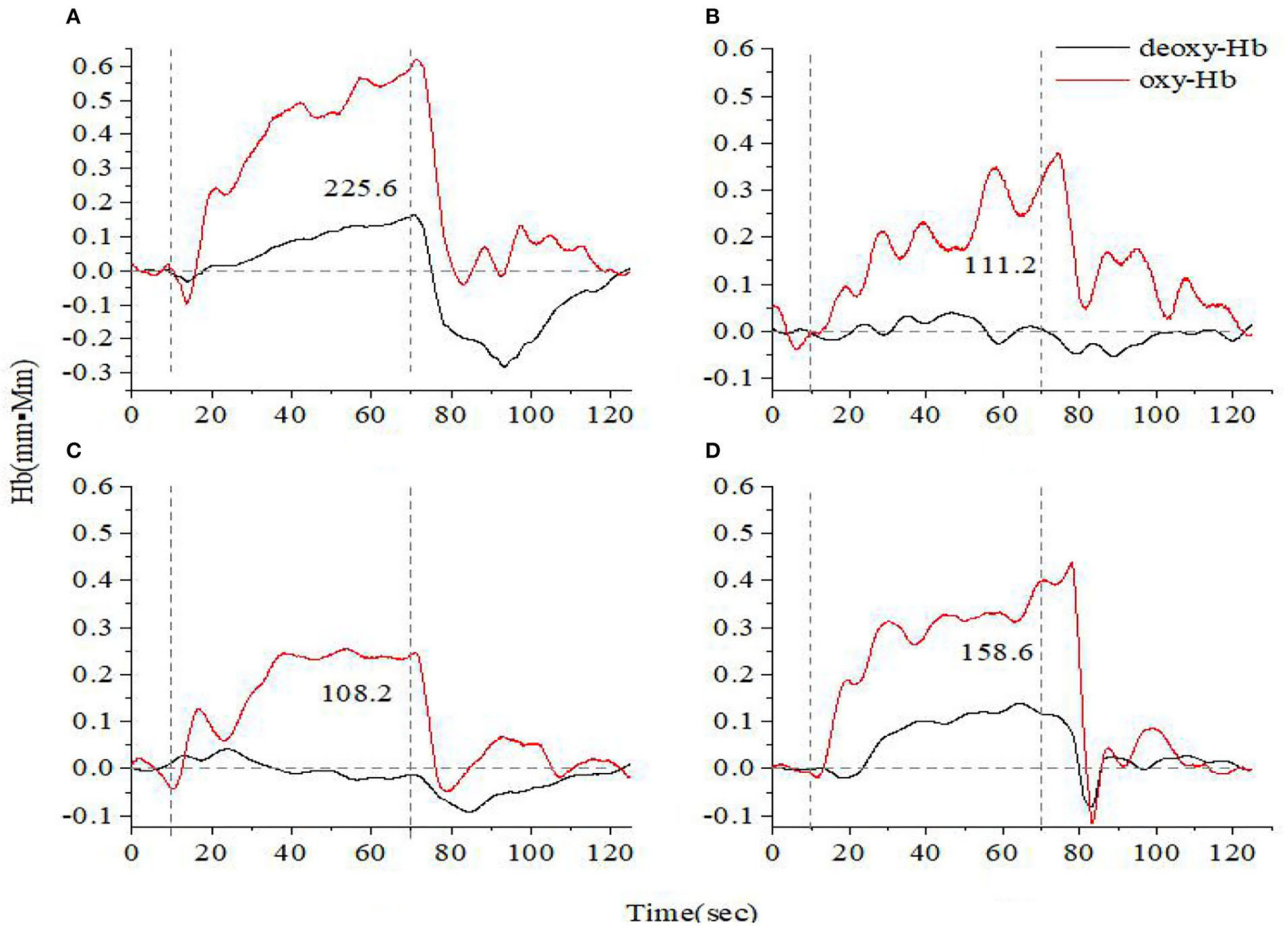
Time course of hemoglobin concentration changes in a representative healthy control **(A)** and a representative anxiety and depression patient at the acute **(B)**, consolidation **(C)**, and maintenance stages **(D)**. All of them completed the high-level cognition task. The red line indicates the amount of change in oxyhemoglobin concentration (oxy-Hb), and the black line indicates the amount of change in deoxyhemoglobin concentration (deoxy-Hb). The numbers indicate integral values.

Figures 1, 2 show the prefrontal oxygen response of A&D at different stages and a HC during the VFT and HCT. It can be seen that the concentration of oxy-hemoglobin responded more significantly to the task, increasing as the task is being performed and reaching a peak value at the end of the task. Thereafter, oxy-Hb gradually returned to baseline values. By contrast, the deoxy-hemoglobin concentration changed much less and with more fluctuations.

Additionally, we found that the oxy-Hb recovered more rapidly toward the baseline under the HCT paradigm than that under the VFT paradigm, which is anticipated. As mentioned earlier, the HCT generates more intensive stimulus to the brain cortex, activating more oxy-Hb response. Hence, it is reasonable to see the fast and sharp decrease when the HCT test stopped, leading to a fast recovery to the baseline.

For both VFT and HCT, the oxy-Hb response was generally lower in A&D than in HCs when performing the same task. Besides this, as shown in Figures 1, 2, for the same A&D patient, the HCT produced larger oxy-Hb integral values, indicating a higher activation of the prefrontal lobes in this task. Under the HCT, the difference between normal and acute patients is more obvious compared with the VFT.

**Figure 3:**
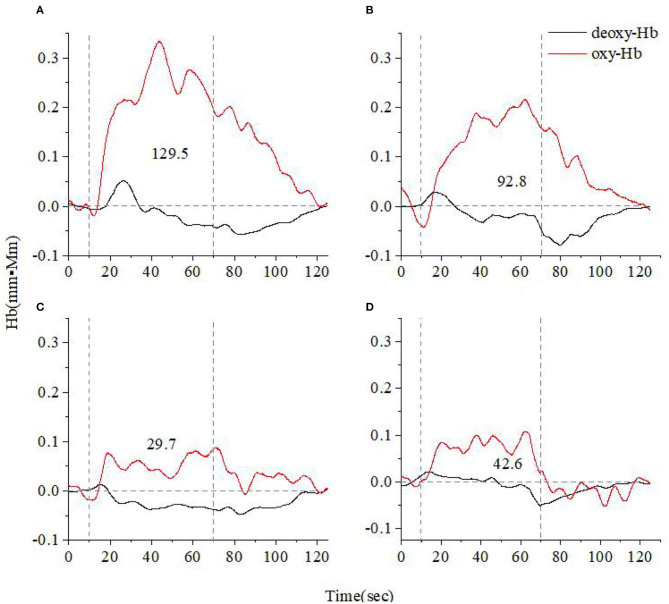
Time course of the average hemoglobin concentration of 10 people under verbal fluency task (VFT) **(A)**. Time course of the average hemoglobin concentration of 10 anxiety and depression patients at the acute **(B)**, consolidation **(C)**, and maintenance stages **(D)**. All of them completed the VFT. The red line indicates the amount of change in oxyhemoglobin concentration (oxy-Hb), and the black line indicates the amount of change in deoxyhemoglobin concentration (deoxy-Hb). The numbers indicate integral values.

**Figure 4:**
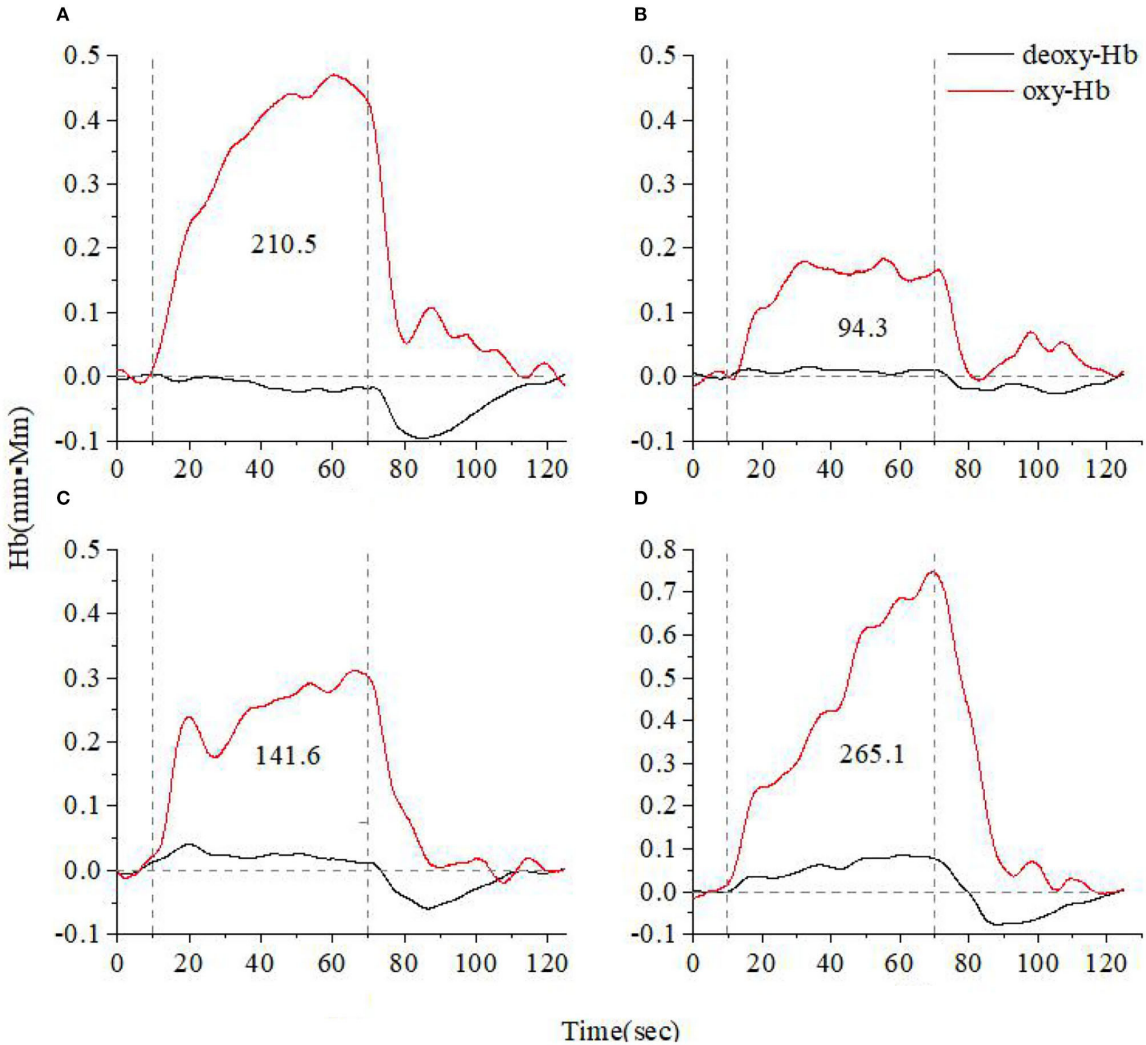
Time course of the average hemoglobin concentration of 10 people under verbal fluency task **(A)**. Time course of the average hemoglobin concentration of 10 anxiety and depression patients at the acute **(B)**, consolidation **(C)**, and maintenance stages **(D)**. All of them completed the high-level cognition task. The red line indicates the amount of change in oxyhemoglobin concentration (oxy-Hb), and the black line indicates the amount of change in deoxyhemoglobin concentration (deoxy-Hb). The numbers indicate integral values.

Figures 3, 4 show the average cerebral oxygen responses over A&D at different stages and HCs under the VFT and HCT, respectively. From the comparison, it is clear that the average responses of the HCs and A&D are similar to those of the representative subjects, i.e., the activation by HCT is greater than that by VFT. In addition, for either task, the hemodynamic response was much greater in HCs than in A&D. Nevertheless, the difference between the acute phase A&D patients and healthy controls in hemodynamic response (oxy-Hb and deoxy-Hb) was not significant under VFT (*p* > 0.05).

### Activation Response to the Tasks in Patients at Different Stages

**Figure 5:**
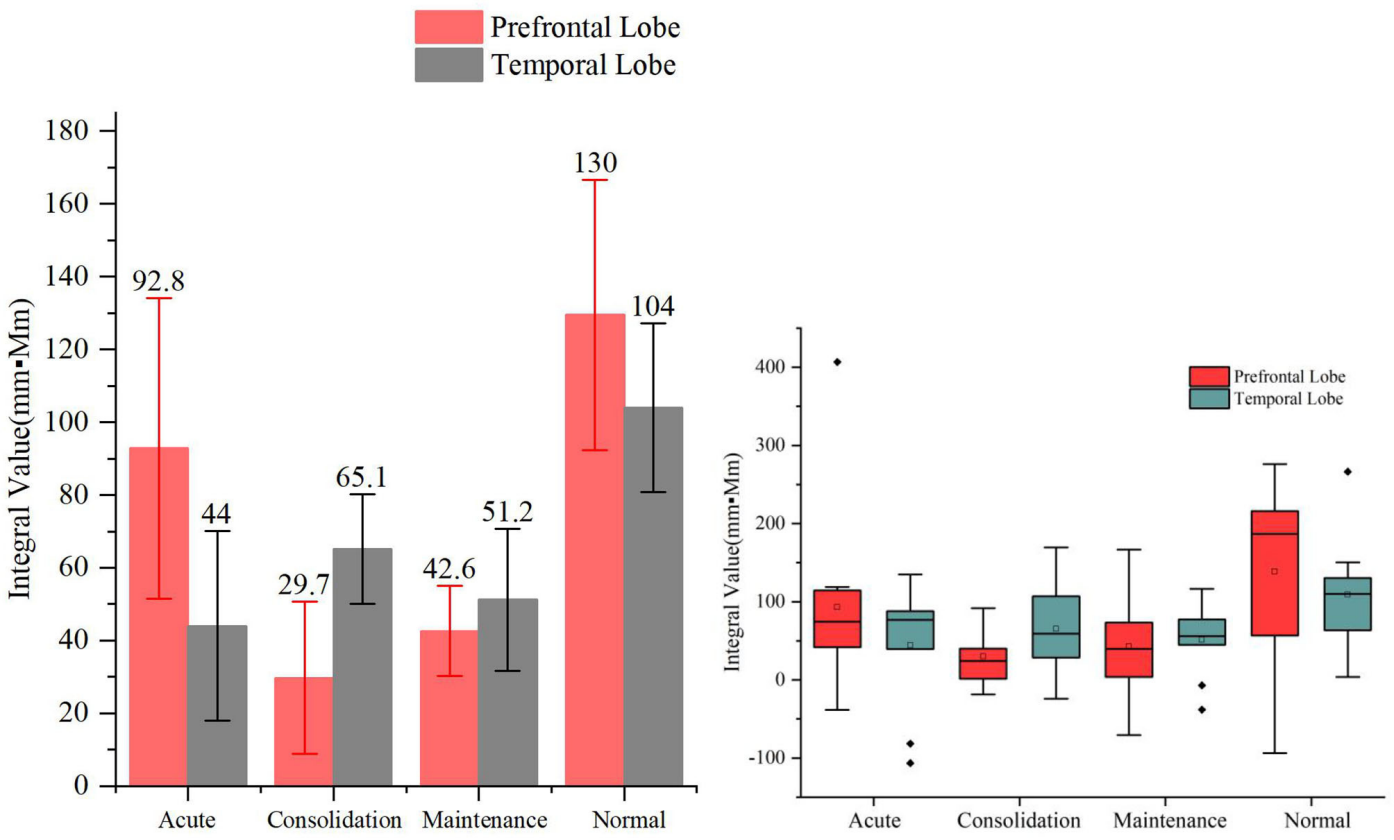
Integral values (mean ± SD) of prefrontal and temporal oxy-Hb from the anxiety and depression patients at the acute, consolidation, and maintenance stages as well as the healthy controls during the verbal fluency task. No significant differences were found in the oxy-Hb integral values among the four groups.

As shown in Figure 5, the acute, consolidation, and maintenance stages of A&D have, on average, a lower activation than that of healthy controls by the VFT, but the difference between these groups was not significant (*p* > 0.05). In addition, the prefrontal and temporal lobe responses were not always consistent at different stages, especially when the hemodynamic response was low.

When HCT was used as the activation task, we found that the hemodynamic responses differed significantly across the different stages of A&D in the prefrontal lobe. The stages with oxy-Hb integral value from the largest to the smallest are consistent, i.e., in order—maintenance stage, consolidation stage, and acute stage. The integral values are consistent with the clinical symptoms. For example, the patients had the lowest mean hemodynamic response at the acute stage, an enhanced response at the consolidation stage, and the largest mean response at the maintenance stage, which was slightly larger than that of the healthy population on average. The cerebral oxygen activation in the temporal lobe at the different stages of A&D was consistent with the results in the prefrontal lobe.

However, we also found that the integral values within each group had a large variation, resulting in statistically insignificant differences (i.e., *p* > 0.05) between groups. Nevertheless, we found significant differences in temporal lobe integral values between A&D at acute stage and HCs when utilizing the HCT (*p* < 0.001). Besides this, the other significant tests among the four groups are marginal (i.e., the *p*-value is slightly larger than 0.05). Overall, the group differences under HCT were much better than that under the VFT.

The oxy-Hb of the VFT test is higher in the acute and the normal groups in the prefrontal than the temporal lobe. The HCT shows that all of them has higher oxy-Hb in the prefrontal lobe. The frontal lobe is mainly responsible for higher-level thinking, and the temporal lobe is responsible for memory and processing of information. The frontal lobe has a stronger cognitive function than the temporal lobe, so the oxy-Hb of the frontal lobe is higher than that of the temporal lobe. At the same time, HCT intensity was higher than that of VFT, so oxy-Hb was higher under HCT.

As mentioned earlier, the oxy-Hb integral values of the A&D patients at the maintenance period is slightly higher than those of healthy controls. Nevertheless, no significant difference was found between the two groups, which coincides with previous reports (33) and might be due to the small sample size or large individual variability.

## Discussion

We consider the HCT as a comprehensive capability of the human's advanced thinking and performance, such as integrating and processing concepts, making judgment, reasoning as well as executing to solve complicated problems. These advanced thinking and performance would generate more brain cortex activation when compared with the VFT that only involves simple thought and immediate response. Thus, the details of HCT vary a lot among different studies, and there are no standard procedures. Some studies used Stroop tasks and the Tower of London tasks (34–36). The patients' language memory, working memory, reaction speed, attention, processing speed, and executive function were measured. The emotion recognition tasks are used to evaluate executive function and reflect the subjects' various types of abilities, such as complex visual and spatial planning, working memory, and selective attention (37–39), which reflect the patient's executive functions, attention, discrimination, memory, and cognitive control. All of these tasks involve advanced thinking and performance. Hence, we defined these activities as “high-level cognition task” in this study and investigated the impact of HCT on oxygenation activation of the brain cortex.

This study confirms that HCT generates more intensive stimulus to the brain cortex, activating more oxy-Hb response. However, VFT only covers limited executive functions, such as word extraction ability, which are not enough to distinguish different psychosis (40). According to the theory of brain function (22, 41, 42), the frontal lobe is mainly responsible for higher level thinking, and the temporal lobe is responsible for cognition and information processing. The frontal lobe has a stronger cognitive function than the temporal lobe.

The main objective of this study was to evaluate the task intensity for the frontal and temporal cortex activation paradigm in A&D by using the fNIRS technique. The VFT and HCT were adopted to perform cortex excitation. Among these, VFT is an established task and more commonly employed for fNIRS brain function assessment. For example, Herrmann et al. (43, 44) applied the VFT to psychiatric patients, and the fNIRS outcomes found remarkable hemoglobin changes in patients with depression during the task, indicating the cerebral function alternations in this population. The observation of these changes may provide insight into clinical diagnosis and guide interventions (45).

We recruited A&D at different stages (acute, consolidation, and maintenance) for task evaluation and compared them with age-matched HCs. The integral value of oxy-Hb during the task was used to represent the total amount of cortex activation. Individual and group-averaged cerebra oxygen responses showed that A&D generally had less cortex activation during the task compared to HCs (Figures 1–4). In both HCs and A&D, the HCT had a larger integral value of oxygen response than the VFT, indicating that the HCT is more intensive in activating the prefrontal and temporal lobes than the VFT. Thus, HCT can better reflect the function of the cerebral cortex. The possible reason is that the HCT is more challenging and would trigger a comprehensive response of human cognitive capacity. The reports from other research groups also support this explanation (46, 47).

**Figure 6:**
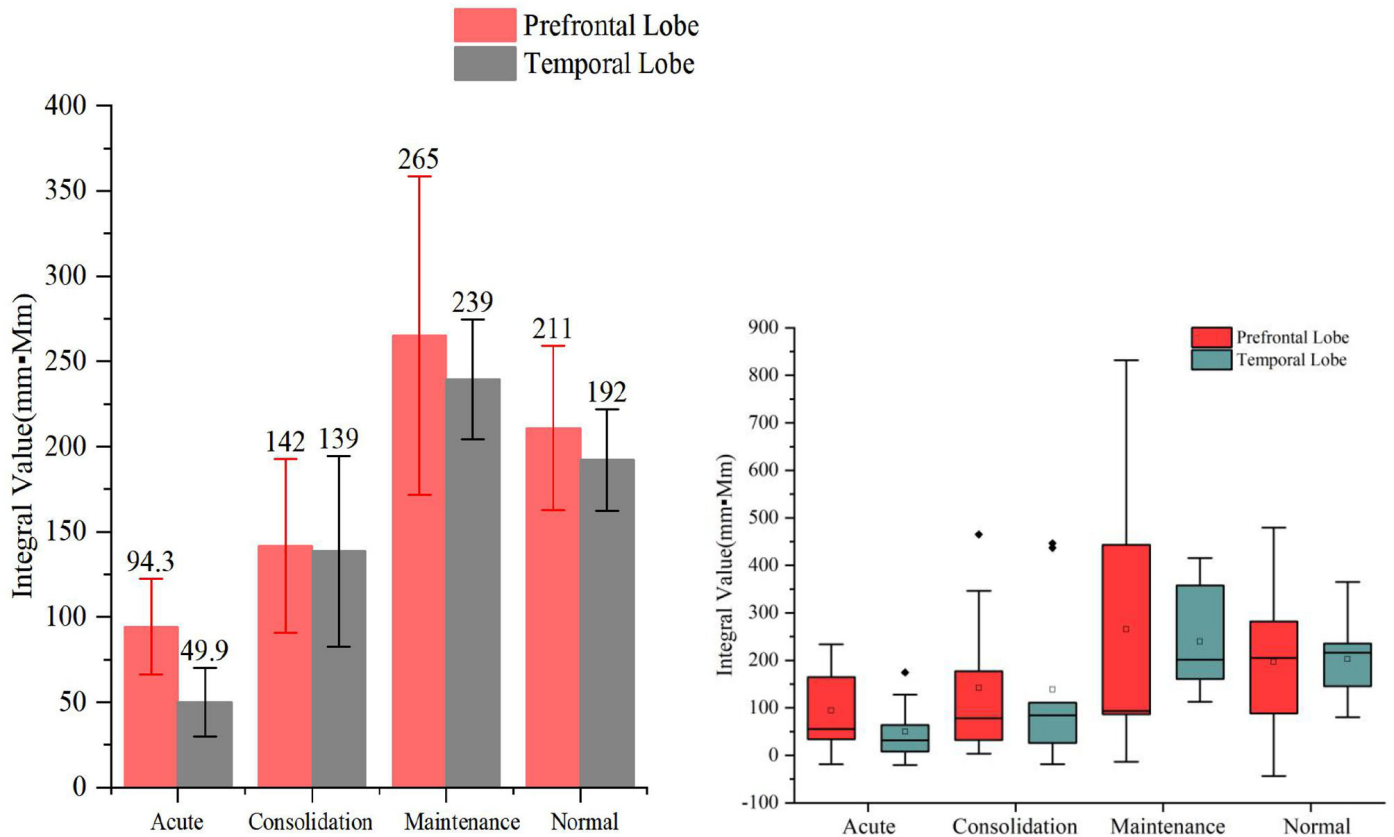
The integral values (mean ± SD) of prefrontal and temporal lobe oxy-Hb from the anxiety and depression patients at the acute, consolidation, and maintenance stages as well as the healthy controls (HCs) under the high-level cognition task. The oxy-Hb integral values of both temporal lobes of the patients at the acute stages were significantly smaller than those of the HCs' (*p* < 0.001).

Despite the relatively weak activation by the VFT, we found that the cerebral oxygen responses in A&D were generally lower than in HCs under this paradigm (Figures 5, 6). However, the difference in blood oxygenation among the different stages of A&D was not significant, indicating that the VFT is not suitable as a stage marker. This may be due to the fact that the VFT does not sufficiently challenge the subjects' information processing abilities. By contrast, the HCT efficiently stimulates the advanced cognitive abilities in the human cerebral cortex. Although individual variability was found among subjects in the same group, A&D showed different responses at different stages. The response was lowest especially at the acute stage, then it was enhanced during the consolidation stage and ultimately became strongest during the maintenance stage, which is close to that of healthy individuals.

We performed the first evaluation of the HCT using the fNIRS technique, and the fNIRS measurement could detect a cognitive impairment of advanced function that is associated with A&D in a timely manner. Furthermore, the HCT would be helpful to predict the disease progression of A&D and identify the stage when integrated with clinical symptoms as assessed by HAMD-24 and HAMA-14. When the symptoms are relieved, the patients' advanced cognitive function can be partially restored to a pre-disease normal level, which provides an objective basis for the assessment of treatment effects.

Although we found an oxygen response difference between A&D and HCs, either individually or by group, statistical analyses reveal that the oxygen responses varied substantially within any group. Therefore, the significant differences in integral values were found only between A&D at the acute stage and HCs in the temporal lobe region (*p* < 0.001) when using the HCT. By contrast, no significant differences were found between any groups when using the VFT. The statistical outcomes might be affected by both the intensity and the duration of the task activating the cerebral cortex. Under the weak activation paradigm, cerebral oxygen changes associated with cognition are easily buried by baseline perturbations, leading to the outcome of non-significant differences. The frontal and temporal cortex task protocol needed to be further enhanced to better differentiate between healthy and psychosis.

## Conclusions

To conclude, the VFT generates a weaker cortex activation when compared with the HCT. Our study showed that HCT was associated with A&D and more efficient in differentiating the patients from healthy controls than the VFT. Moreover, the cerebral oxygen responses are related to progresses of A&D; thus, it could be used as a diagnostic tool to identify A&D and distinguish disease stages. In future studies, we will recruit more patients with different types of psychosis (e.g., depression, anxiety, and schizophrenia), with the aim to evaluate the comprehensive cognitive impairments by using the HCT paradigm.

## Limitation

The relatively small size (i.e., 10 samples in each group) is the main study limitation. In future studies, first of all, we will enlarge the sample size to draw more solid conclusions. Second, we will focus on the longitudinal experimental design, collecting the cognitive status of two time points (baseline and follow-up), and carrying out cognitive training, such as abstract thinking training. Lastly, we should take into account pre-disease IQ (48), cognitive reserve, and the prevalence of cognitive impairment (49–51). We aim to compare how sensitive the oxygen response is to the HCT and VFT, which has not been reported in previous studies. Optimization of the HCT paradigm for diagnosis of psychiatric diseases will be our future work.

## Data Availability Statement

The original contributions presented in the study are included in the article/supplementary material, further inquiries can be directed to the corresponding author/s.

## Ethics Statement

The studies involving human participants were reviewed and approved by the Ethics Committee of the First Hospital of Shanxi Medical University. Written informed consent to participate in this study was provided by the participants' legal guardian/next of kin. Written informed consent was obtained from the individual(s), and minor(s)' legal guardian/next of kin, for the publication of any potentially identifiable images or data included in this article.

## Author Contributions

YX conceived of and led on the study design. DW and XL managed the literature searches. DW undertook the statistical analysis, under the supervision of YX. DW and XL wrote the first draft and the subsequent revisions of the manuscript. QL, QY, and MW contributed to collecting data. All the authors contributed to and have approved the final manuscript.

## Conflict of Interest

The authors declare that the research was conducted in the absence of any commercial or financial relationships that could be construed as a potential conflict of interest.
